# Comparing Nutrient Removal from Membrane Filtered and Unfiltered Domestic Wastewater Using *Chlorella vulgaris*

**DOI:** 10.3390/biology7010012

**Published:** 2018-01-19

**Authors:** Elyssia Mayhead, Alla Silkina, Carole A. Llewellyn, Claudio Fuentes-Grünewald

**Affiliations:** Bioscience department, Swansea University, Singleton Park, Swansea SA2 8PP, UK; elyssia.mayhead@gmail.com (E.M.); a.silkina@swansea.ac.uk (A.S.); c.a.llewellyn@swansea.ac.uk (C.A.L.)

**Keywords:** domestic wastewater, bioremediation, membrane technology, microalgae, *Chlorella vulgaris*, eutrophication

## Abstract

The nutrient removal efficiency of *Chlorella vulgaris* cultivated in domestic wastewater was investigated, along with the potential to use membrane filtration as a pre-treatment tool during the wastewater treatment process. *Chlorella vulgaris* was batch cultivated for 12 days in a bubble column system with two different wastewater treatments. Maximum uptake of 94.18% ammonium (NH_4_-N) and 97.69% ortho-phosphate (PO_4_-P) occurred in 0.2 μm membrane filtered primary wastewater. Membrane filtration enhanced the nutrient uptake performance of *C. vulgaris* by removing bacteria, protozoa, colloidal particles and suspended solids, thereby improving light availability for photosynthesis. The results of this study suggest that growing *C. vulgaris* in nutrient rich membrane filtered wastewater provides an option for domestic wastewater treatment to improve the quality of the final effluent.

## 1. Introduction

The exponentially increasing human population has led to intense consumption of freshwater through domestic, agricultural and industrial uses, generating large volumes of wastewater [1]. A total of 66% of the world’s population is predicted to live in urban areas by 2050 [2]; this will place increased pressure on the finite freshwater resource and requires efficient domestic wastewater management to keep up with demand, whilst mitigating the global environmental phenomenon: eutrophication [3,4]. However, there are a number of issues with wastewater treatment that need to be addressed in order to sustainably improve the quality of effluent discharged from treatment facilities. Issues include: high energy consumption, particularly in biological treatment of domestic wastewater and the use of chemicals to control high nutrient effluent levels which have economic and environmental costs [5]. Loading of bioavailable nutrients and degradable organic material into aquatic systems poses another issue due to the resulting environmental degradation of rivers, lakes and coastal water bodies [6,7]. The main objective of domestic wastewater treatment is to remove or decrease: (1) harmful levels of microbes (e.g., pathogenic coliform bacteria, protozoa, viruses and parasitic worm eggs), (2) biochemical oxygen demand (BOD), (3) pollutant levels of nutrients (e.g., nitrogen (N) and phosphorus (P)), (4) heavy metals (e.g., zinc, copper, mercury, lead, aluminium) and (5) solid material [8,9].

**Figure 1:**
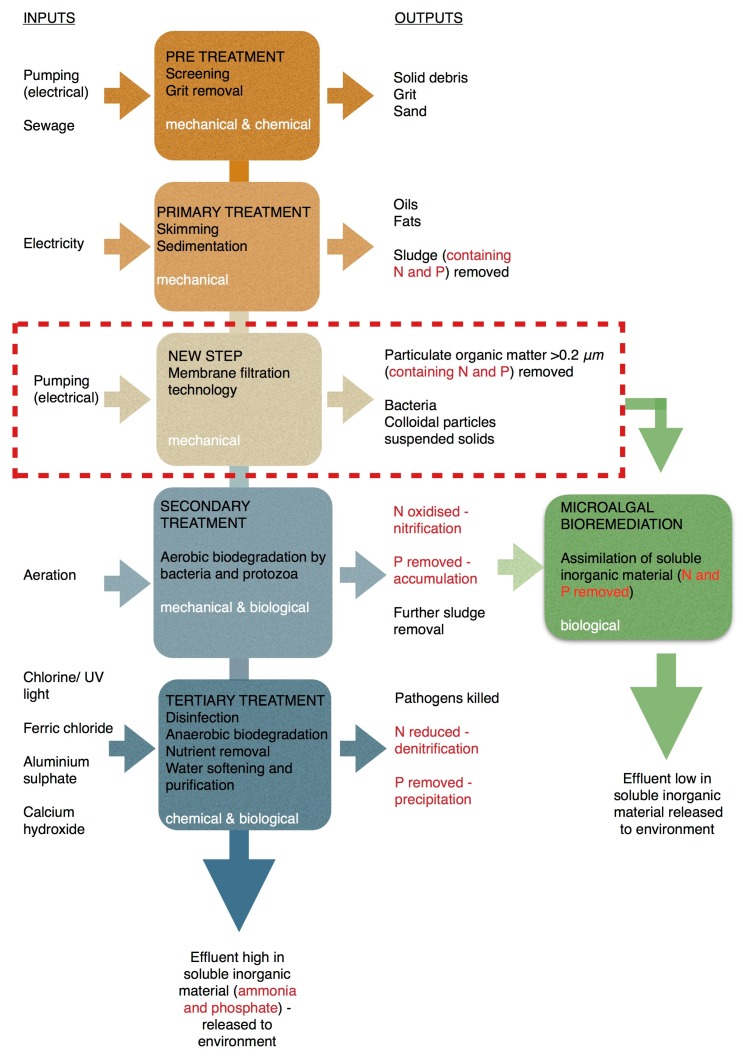
Generalised wastewater treatment process and proposed technology. Figure adapted from Carlisle [13].

Current wastewater treatment occurs through a number of stages including pre-treatment, primary (mechanical), secondary (biological) and in some cases tertiary (chemical) treatment, as shown in Figure 1 [10]. European regulations require wastewater to be treated to at least the secondary treatment level [11]. This level of treatment ensures removal of BOD, preventing depletion of dissolved oxygen in receiving water bodies and resultant death of aerobic organisms such as fish. Harmful microbes are reduced to safe levels, primarily through the process of biological oxidation and adsorption [8,12].

Despite the process of primary and secondary wastewater treatment, wastewater effluent can remain loaded with soluble inorganic pollutants such as NH_4_-N_,_ PO_4_-P and heavy metals due to the inability of bacteria and protozoa, the predominant microbial community, to assimilate them [12]. Therefore, wastewater treatment facilities that discharge effluent into sensitive areas (as classified by the Water Framework Directive (WFD)) operate tertiary treatment as a means of removing these soluble compounds [8,14]. However, the complete tertiary process has been estimated to be four times more expensive than primary treatment and requires toxic chemicals [15]. These challenges present the potential for more innovative approaches to wastewater treatment. One innovative approach for removal of soluble inorganic pollutants from domestic wastewater is through bioremediation, using naturally occurring photosynthetic microalgae [8,16,17], a process otherwise known as phycoremediation. Microalgae are superior to other microorganisms in bioremediation of wastewater as they are able to acquire inorganic compounds far more readily than bacteria. Microalgae also have reduced health and safety risks associated with accidental release to the environment, as they are relatively non-pathogenic [18]. Phycoremediation of wastewater has the potential to reduce the use of harmful chemicals such as aluminium sulphate and ferric chloride, currently used to remove soluble phosphate (PO_4_-P) [19]. However, optimum culture conditions are essential for effective phycoremediation. Therefore, low energy physical treatment such as filtration by pressure driven membrane technology has been proposed and can offer a sustainable low-cost solution. Membrane Technology (MT) is scalable with numerous arrangements and alternatives and easy to incorporate and integrate into waste treatment processes. It offers low operational cost (OPEX) compared to other competing technologies since there is no phase change required and minimal or no use of chemical additives [20,21]. Using this technology, waste can be recycled back to the production systems substituting for newly manufactured materials.

In this study, MT has been applied to the domestic wastewater treatment process in order to improve the culture conditions for microalgal bioremediation. Through improving the culture conditions and subsequent nutrient removal efficiency of microalgae at the tertiary treatment stage, MT has the potential to reduce the cost of the whole domestic wastewater treatment process. MT has been used in water purification and wastewater treatment since the late 1960’s. Microfiltration, in particular, can be used to remove unicellular bacteria and protozoa that feed on, compete with or inhibit microalgae [22]. Membrane filtration is therefore being considered as an alternative to UV radiation in removing bacteria and purifying water in Korea [23]. Colloidal particles and suspended solids are also removed by microfiltration, and studies have revealed that removal of these components of waste streams increases light utilization in photosynthesis, which improves microalgal growth and subsequent inorganic nutrient removal [21,23]. Previous research [20,21,22,23,24] has demonstrated that membrane filtration integrated in the wastewater treatment cycle has been proposed and successfully applied for converting waste sludge into a particle free, nutrient rich fluid and a nutrient depleted solid stream. The solid fraction is a retentate stream with reduced nutrient content that can be applied to land as an organic fertilizer, while the soluble organic matter (ammonia and phosphate) can be concentrated and formulated into more useful materials and so valorising this route for the waste. The soluble fraction is a source of water and nutrients used as growth media for cultivation of algae, microbes and plants. Microalgal cultivation uses nutrients such as Nitrogen (ammonium) and Phosphorus (ortho-phosphate) for the growth of valuable algal biomass. The remediation process is very quick and represents environmentally friendly scalable technology, as does MT. These two technologically low cost processes could be applied together for successful waste remediation.

The aims of this study were to:

(1) Evaluate the application of microalgae to current wastewater treatment with a focus on improving the quality of final effluent.
(2) Assess the use of membrane technology to be included as a part of the wastewater treatment process.

## 2. Materials and Methods

### 2.1. Wastewater Collection

Primary domestic wastewater (ww) was obtained from a Welsh Water treatment facility in Southgate, Swansea (51°34′12″ N, 4°05′01″ W). At this facility, sewage undergoes primary and secondary treatment. A thirty-litre aliquot of primary wastewater was a grab sample from the settling tank prior to biological filtration [25]. Samples were transported to the Centre for Sustainable Aquatic Research located in Swansea University, South Wales, and stored at 3 °C for one week until sterilisation.

### 2.2. Pre-Treatments and Characteristics of Wastewater

The wastewater was sterilised (10 L at a time) following Li et al. [26] and Sharma et al. [27] using an autoclave oven at 121 °C for 20 min (Rodwell model 56, Rodwell, Basildon, UK). The pre-treatment of wastewater used in the filtered Treatment 1 underwent microfiltration using a membrane filtration rig with a 0.2 μm pore size hollow fiber (polysulfone) tangential flow cartridge (GE healthcare); the active surface area in the cartridge was 0.9 m^2^, and the average cross flow was 778 L h^−1^. The nutrient concentration (NH_4_-N; PO_4_-P) of the sampled wastewater is shown in Table 1.

### 2.3. Experimental Layout

#### 2.3.1. Algal Species and Culture Conditions

The unicellular green microalgae species *Chlorella vulgaris* Beijerinck (1890) strain CCAP 211/11R was cultivated under a controlled temperature of 20 ± 1 °C using four conditions carried out in triplicate [26,28,29]. The three conditions were:

**a**  (Control) F2P media in freshwater
**b**  (T1) 100% primary filtered ww
**c**  (T2) 100% primary unfiltered ww

The F2P medium provides known optimal culture conditions for microalgal growth and nutrient uptake. Therefore, F2P was used as a control in this experiment to enable comparison with the results of wastewater cultivation. T1 was used for comparison with T2 to investigate the effect of membrane filtration on microalgal growth and nutrient uptake in primary ww. 

#### 2.3.2. Bubble Column System

**Figure 2:**
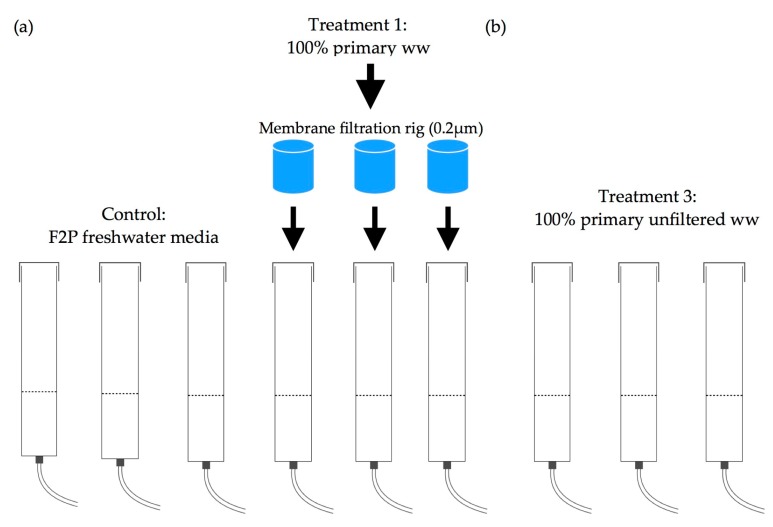
Bubble columns system for *Chlorella vulgaris* cultures (**a**) Experiment one, (**b**) Experiment two.

*Chlorella vulgaris* was cultivated autotrophically by batch culture in sterilised bubble columns of 0.3 cm thick transparent acrylic (height: 120 cm, internal diameter: 10.5 cm). The study was split into two experiments, each of which spanned 12 days, involving six columns in the first experiment and 3 columns in the second experiment (Figure 2). 

#### 2.3.3. Aeration

**Figure 3:**
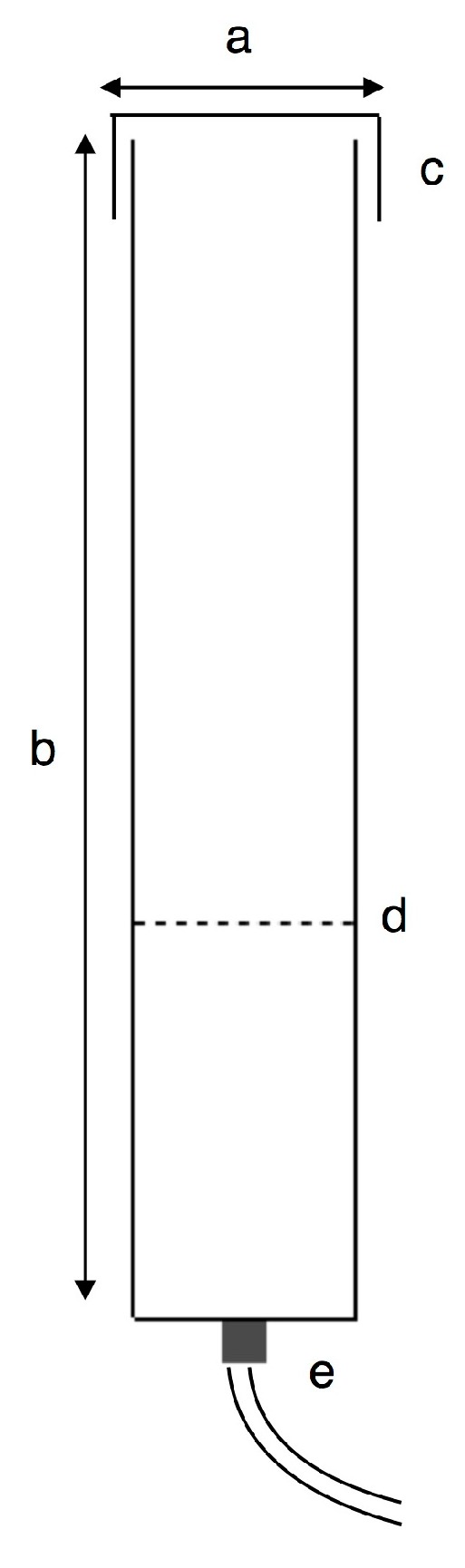
Bubble column apparatus. (**a**) Column diameter 10.8 cm; (**b**) column height 120 cm, (**c**) lid, (**d**) culture level (4 L = 47 cm gas-free liquid height, 3.2 L = 36 cm gas-free liquid height), (**e**) CO_2_ control valve.

Filter sterilized ambient air (0.039% CO_2_) was fed into the bottom of the columns supplied by a pressurized tank and controlled by a valve (Part e. Figure 3). The flow rate was standardized to 0.5 L min^−1^ across columns [28,30]. Aeration with CO_2_ agitated the culture [31]; this was maintained throughout the 12-day period. 

#### 2.3.4. Daily Illumination

The columns were illuminated over an 18 h photoperiod and 6 hr dark period each day. During the photoperiod, white light was provided by tubular fluorescent lamps producing an average of 177 μmol m^−2^ s^−1^ photons (58W Osram, LUMILUX T8, Münich, Germany). Photon flux density was established by taking the mean of three points along columns (n = 6) using a PAR irradiance sensor and Universal Light Meter-500 (WALZ, Effeltrich, Germany). 

#### 2.3.5. Experimental Design

Experiment 1—Control and Treatment 1 (C & T1)

Bubble columns had a working volume of 4 L and gas-free liquid height of 47 cm (Figure 2a). The control consisted of sterilized freshwater and 20 mL cell-hi F2P media (Varicon Aqua Solutions Ltd., Worcestershire, UK) passed through a syringe filter of 0.45 μm (Minisart^®^, Sartorius, Surrey, UK) for sterilization. T1 consisted of 100% membrane filtered primary wastewater. The initial inoculum concentration of *C. vulgaris* taken from a 20 L culture stock was 3.4 × 10^6^ ± 151,905 cell mL^−1^. 

Experiment 2—Treatment 2 (T2)

Bubble columns had a working volume of 3.2 L. The gas-free liquid height was 36 cm (Figure 2b). T2 consisted of 100% unfiltered primary wastewater. The initial inoculum concentration taken from the same 20 L culture stock was 1.4 × 10^6^ ± 283,976 cell mL^−1^, this follows the method of Cho et al. [23]. 

### 2.4. Sampling

Samples (n = 7) were taken at the same time each day (± 2 h) over a period of 12 days. These were taken on consecutive days for the first three samples in both experiments; further samples were taken within a maximum of 72 h of each other. Samples from T2 (unfiltered wastewater) were filtered prior to cell count measurement using a 50 μm mesh, preventing solid particles >50 μm from affecting growth measurements.

### 2.5. Growth Determination

The growth of *C. vulgaris* was monitored by optical density (OD), cell count (CC) and dry weight (DW). Prior to all measurements, samples were mixed for 30 s using a vortex (VWR Vortex Genie^®^, VWR, Lutterworth, UK) to ensure homogeneity. OD was carried out using a spectrophotometer (VWR^®^, version 1200, VWR, Lutterworth, UK) at 750 nm, as this wavelength reduces interference by pigments [32]. To reduce error in measurements above 1.0 absorbance, the sample was diluted by deionised water and the resultant absorption multiplied by the dilution factor. CC was conducted using a phase contrast microscope (Olympus CH series, Olympus, Hamburg, Germany) at ×40 magnification and Neubauer improved haemocytometer following Whitton et al. [33]. Dense cultures (above 100 cells per large square) were diluted with deionised water before counting to reduce human error and to improve accuracy. The centre square of the haemocytometer was used for CC.

DW was conducted using a Millipore Sterifil^®^ filtration system (Merck, Darmstad, Germany) and 0.42 μm glass microfiber filter disks (Whatman^®^ GF/C, GE Healthcare, Maidstone, UK). Filter disks were weighed (KERN ABS80-4N, Kern, Balingen, Germany) prior to filtration. A vacuum pump (Charles Austen model B100, Charles Austen, Surrey, UK) was used to filter a known volume (10 or 20 mL) of culture sample through the filter disk. The resultant filtered water (10 mL) was frozen at −21 °C for later nutrient analysis before washing with 20 mL deionised water, leaving the biomass on the disk. Disks were dried at 80 °C for a minimum of 24 h before taking the final weight of the filter and culture. 

Equation (1) was used to calculate the biomass production as the dry weight of each culture sample:

(1) $$\mathrm{Dry}\;\mathrm{weight}\;\left({g\;L}^{-1}\right)=\frac{\mathrm{Culture}\;\left(g\right)\times 1000}{\mathrm{filtered}\;\mathrm{volume}\;\left(\mathrm{mL}\right)}$$

Equation (2) represents the linear relationship between optical density (750 nm) and dry weight (mg L^−1^):

(2) $$\mathrm{Dry}\;\mathrm{weight}\;\left({\mathrm{mg}\;L}^{-1}\right)=0.275\times OD_{750}-0.0128,\;R^{2}=0.9856$$

Specific growth rate, μ (d^−1^), was determined using Equation (3):

(3) μ (d−1)=ln[x2x1]t2−t1

where *x*_1_ and *x*_2_ are defined as optical density (750 nm) at time *t*_1_ and *t*_2_, respectively. 

### 2.6. Nutrient Analysis and pH Monitoring

Nutrient analysis was conducted for all four conditions at six points during the growth of *C. vulgaris.* For consistent measurement of the depletion of soluble inorganic ammonium (NH_4_-N) and ortho-phosphate (PO_4_-P), photometric analysis was performed. Commercially available colorimetric assay kits (Machery-Nagel NANOCOLOR^®^ ammonium 100 and ortho-phosphate, Machery-Nagel, Düren, Germany) were used for analysis of blue indophenol (585 nm) and phosphomolybdenum blue (690 nm), respectively, using a VWR^®^ V-1200 spectrophotometer.

Changes in pH were monitored using the Aqua Digital™ pH controller (version 201, Aqua Digital, Southhampton, UK). The probe was taken out of the pH 7 buffer solution, and excess solution was removed before placing the probe in each sample for measurement. Between measurements, the probe was cleaned with deionised water, excess was removed, and the probe was placed back into the buffer solution.

### 2.7. Statistical Analysis

All data from bubble columns of both experiments in the study were tested for normality using the Kolmogorov-Smirnov goodness-of-fit test. The assumptions were satisfied, resulting in the use of nested/hierarchical ANOVA where condition was nested within day, which was nested within experiment. Statistical analysis was conducted to determine the significance of the difference between conditions at *p* < 0.05 level, using Minitab 17 (Minitab, Coventry, UK).

## 3. Results

### 3.1. Algal Growth

**Figure 4:**
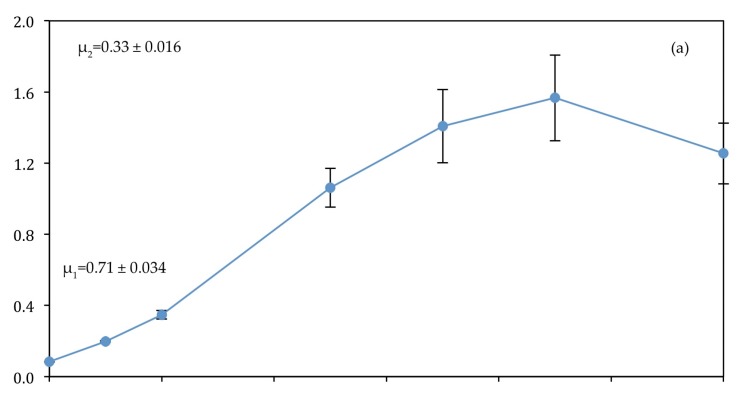

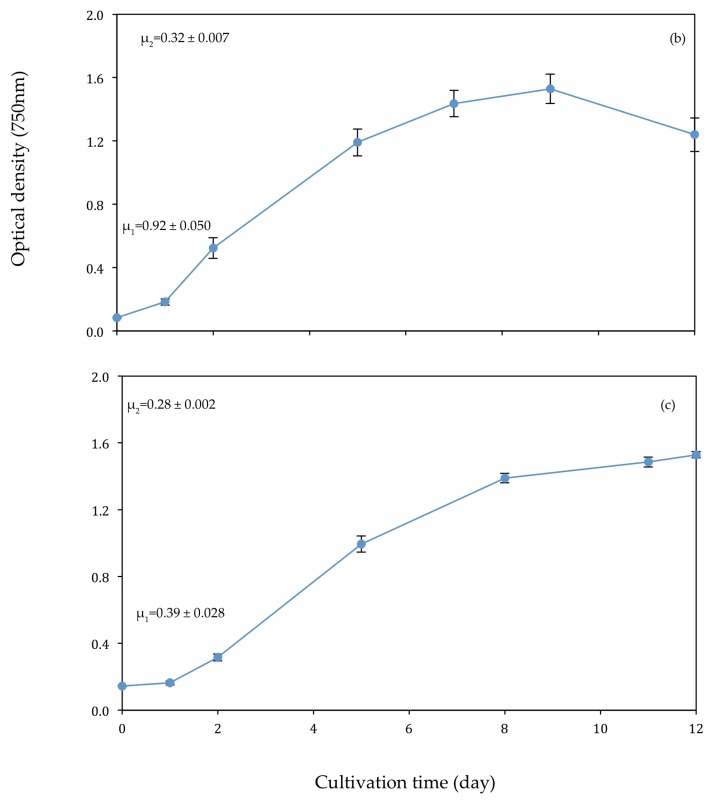
(**a**) Control (**b**) T1 (**c**) T2. Growth curves for *C. vulgaris*. Bars represent standard deviation (SD). Absence of a bar indicates negligible SD. μ_1_ = initial growth rate ± 1 SD, μ_2_ = overall growth rate ± 1 SD. Statistical analysis of the difference in specific growth rates across conditions could not be conducted; however, values for optical density differ significantly between conditions when analysed by nested ANOVA (*f* = 30.515, *p* = 0.005), suggesting a significant difference in growth across conditions.

In the first experiment, *C. vulgaris* underwent a stationary phase beyond day 9. In the Control and T1, this stationary phase is presented by a decline in optical density beyond day 9 in Figure 4a,b. In the second experiment, *C. vulgaris* continued growing beyond day 9 in T2. All conditions experienced a higher initial growth rate (μ_1_) between day 0 and day 2 than the overall growth rate (μ_2_) between day 0 and day 9.

**Figure 5:**
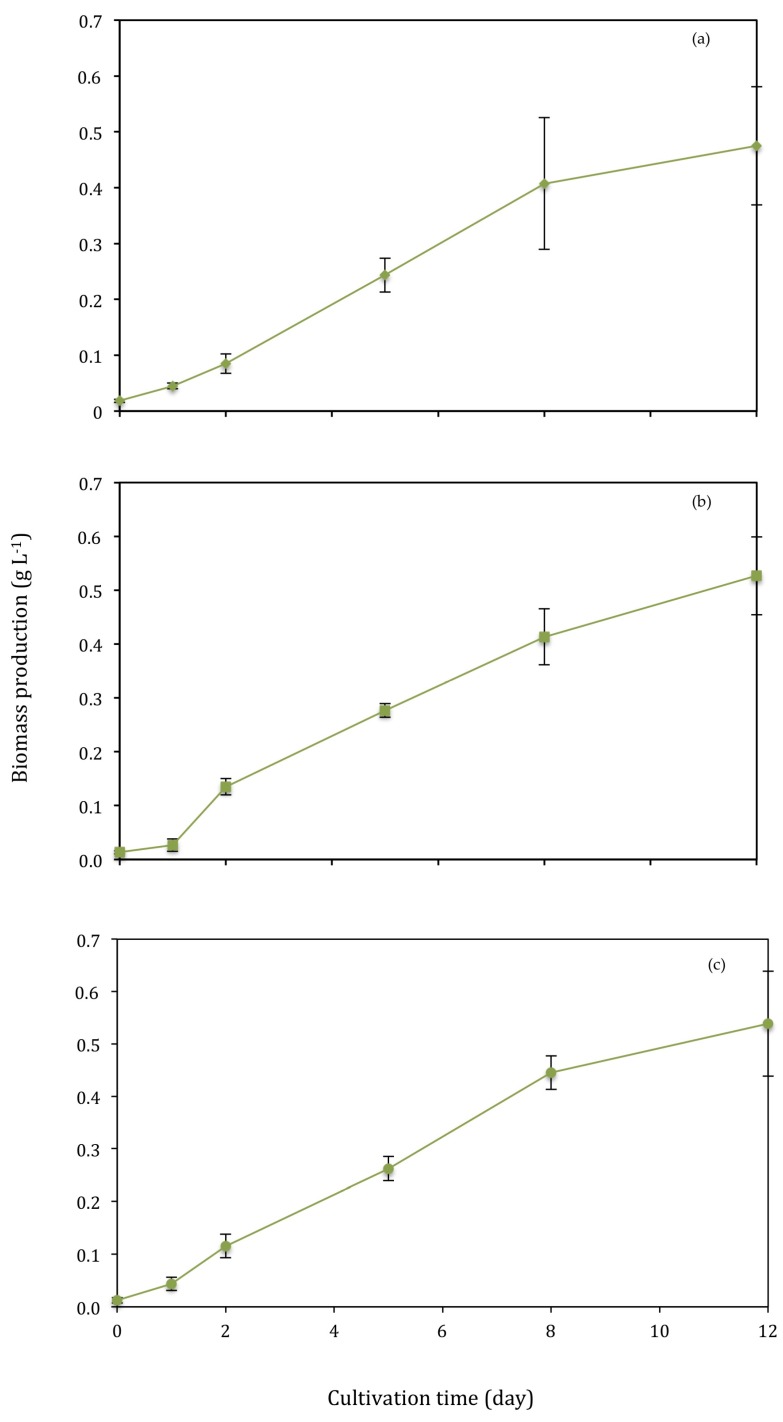
(**a**) Control (**b**) T1 (**c**) T2. Biomass production, determined by dry weight (g L^−1^). Bars represent SD.

T1 experienced the highest initial growth rate (0.920 ± 0.050 d^−1^) (Figure 4). However, the control had the highest overall growth rate of 0.330 ± 0.019 d^−1^. The slopes of the curves suggest that unfiltered wastewater is least suitable for biomass production as this generated the lowest initial and overall growth rates (0.391 ± 0.028 d^−1^, 0.283 ± 0.002 d^−1^, respectively). However, the results of biomass production (Figure 5) do not support this suggestion, as T2 had the highest biomass production of all treatments (0.527 ± 0.104 g L^−1^). The high initial growth rate of T1 coincides with high initial levels of NH_4_-N (85.84 mg L^−1^) and PO_4_-P (13.54 ± 0.16 mg L^−1^), resulting in an N:P ratio of 7.5:1.0.

### 3.2. Biomass Production

T2 had the highest biomass production of all treatments after accounting for the presence of unfiltered particles <50 μm within the samples (Figure 5). Unfiltered particles were accounted for by measuring the dry weight of a sample of unfiltered primary wastewater prior to inoculation with *Chlorella vulgaris*. The average dry weight of unfiltered particles was 0.028 ± 0.007 g L^−1^, which was subtracted from the dry weight measurements for T2 on each of the sample days during the 12-day cultivation period.

Dry weight (g L^−1^) differed significantly (*f* = 212.597, *p* = 0.005) between days, as expected, but not between conditions when analysed by nested ANOVA. Biomass production increased throughout the cultivation period under all conditions (Figure 5). Biomass production of the 12-day cultivation period was 0.457 ± 0.103 g L^−1^, 0.513 ± 0.070 g L^−1^ and 0.527± 0.104 g L^−1^ in the control, T1 and T2, respectively. The F2P media control had a biomass productivity of 0.190 ± 0.048 g L^−1^ d^−1^. The average biomass productivity of T1 and T2 was 0.164 ± 0.020 g L^−1^ d^−1^.

### 3.3. Bioremediation of Nutrients from Wastewater

**Figure 6:**
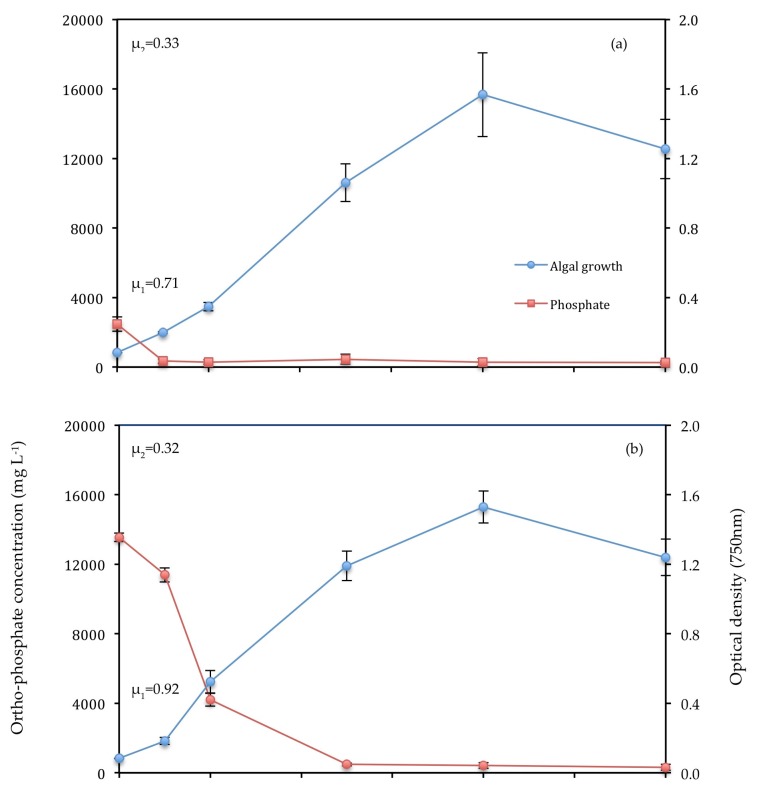

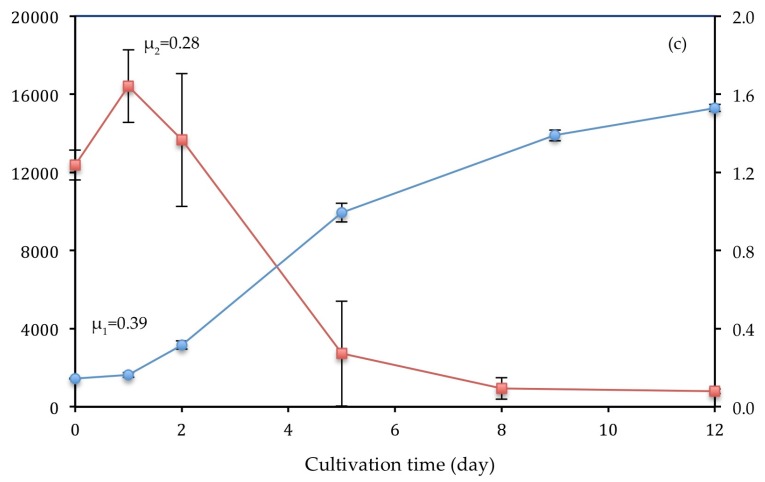
(**a**) Control (**b**) T1 (**c**) T2. Optical density of *C. vulgaris* and depletion of PO_4_-P (mg L^−1^). Bars represent SD. Absence of a bar indicates negligible SD.

**Figure 7:**
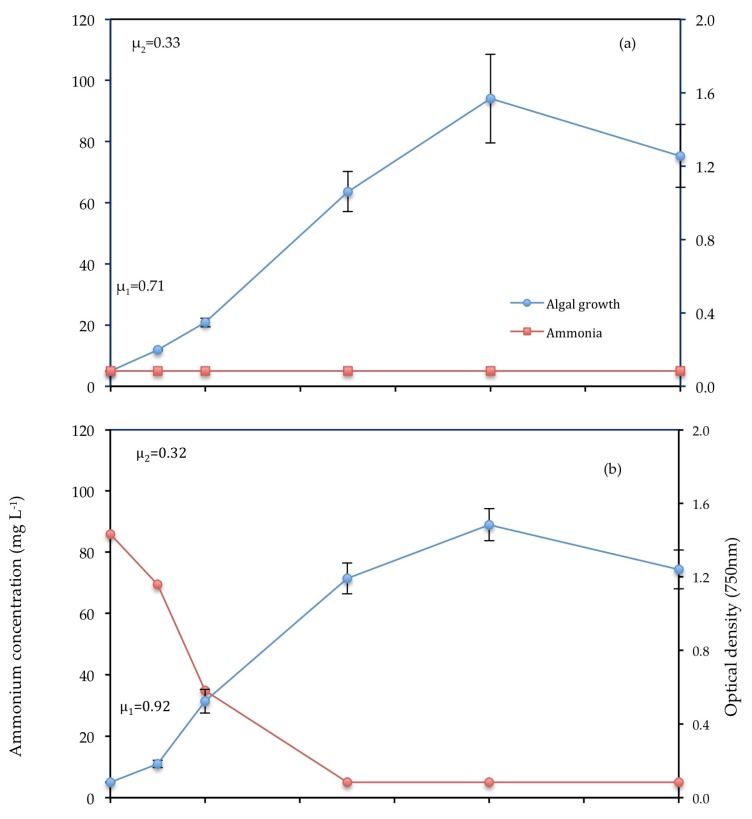

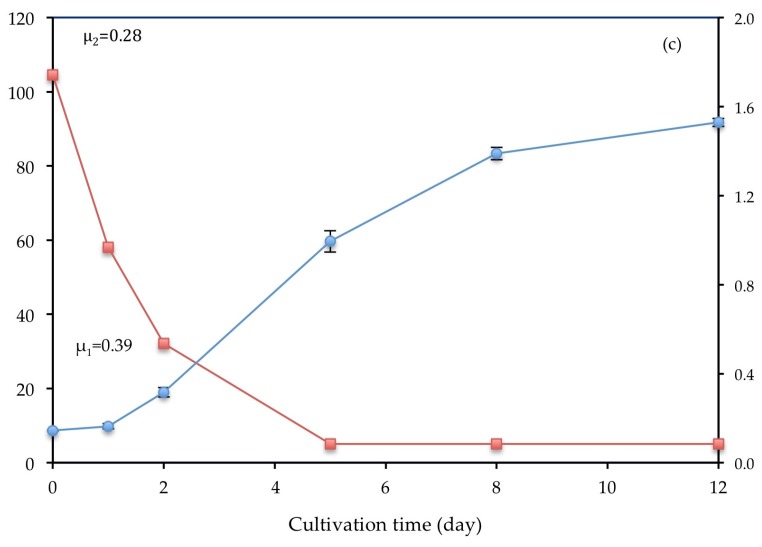
(**a**) Control (**b**) T1 (**c**) T2. Optical density of *C. vulgaris* and depletion of NH_4_-N (mg L^−1^). Bars represent SD for optical density.

NH_4_-N and PO_4_-P concentrations in all of the different wastewater compositions decreased over the cultivation period (Table 2, Figure 6 and Figure 7). There are no comparable measurements for levels of nutrients in the Control because F2P media is composed mainly of nitrate (NO_3_^−^) and chemically bound phosphate (H_2_PO_4_^−^). T2 (unfiltered primary wastewater) consisted of a higher initial level of NH_4_-N and PO^4^-P compared with T1 (filtered primary wastewater). T1 had the highest N:P ratio. Removal of NH_4_-N was most efficient in T2 (95.22%), whereas removal of PO_4_-P was most efficient in T1 (97.69%). Removal percentage was used to determine removal efficiency because large variations occurred in the initial concentration of NH_4_-N and PO_4_-P across conditions.

#### 3.3.1. Ortho-Phosphate

When PO_4_-P was depleted below 0.42 mg L^−1^ in the Control (Figure 6a) and T1 (Figure 6b), algae ceased growth and entered the death phase at 8–12 days. However, PO_4_-P levels did not fall below this point at any stage of growth in T2 (Figure 6c). Figure 6c shows a peak in PO_4_-P on day 1. This is likely to be due to an error in nutrient analysis as no such peak is observed in previous Figure 6a,b. Analysis by nested ANOVA revealed significant difference (*p* < 0.05) between the means of PO_4_-P of different conditions. T1 (filtered primary wastewater) underwent a significant decrease (*f* = 3.322, *p* = 0.003) in PO_4_-P from day 1 to day 2 that did not occur in T2 (unfiltered primary wastewater). PO_4_-P decreased significantly (*f* = 3.322, *p* = 0.003) between days 0 and 5 in Treatments 1 and 2, suggesting that uptake led to rapid growth of *C. vulgaris*.

#### 3.3.2. Ammonium

NH_4_-N was depleted to 5 mg L^−1^ by day 5 in Treatments 1 and 2. Further depletion below this concentration was not detected by nutrient analysis as presented by the consistent low level across conditions from day 5 onwards. There was a decline in NH_4_-N in Treatments 1 and 2 between days 0 and 5 as the optical density of *C. vulgaris* increases. The level of NH_4_-N in the control was too low (<5 mg L^−1^) for detection.

### 3.4. Comparison of the Results of Bioremediation to Current Wastewater Treatment

The standards set by the EC for nutrient loading to aquatic systems have been used to classify the quality of wastewater after bioremediation along with the quality of final effluent produced by the Welsh Water treatment facility at Southgate, Swansea. The results in Table 3 show that bioremediation by *C. vulgaris* produces effluent with a higher classification (moderate-poor) for discharge into rivers.

## 4. Discussion

This study used a common unicellular freshwater species of green microalgae, *C. vulgaris* (Chlorophyta)*,* as it is robust to a wide range of nutrient loading and temperatures but most importantly because of the species’ reported efficiency as a wastewater treatment agent [34,35,36]. *C. vulgaris* has been used in a variety of waters from textile to fish processing wastewater [37,38,39] where it has exhibited high ammonium (NH_4_-N) and phosphate (PO_4_-P) removal rates. However, the species’ natural occurrence in domestic wastewater, in particular, may present an advantage for use in bioremediation of domestic wastewater [40].

### 4.1. Algal Growth

This experiment used a photoperiod of 18:6 h (light:dark) because this is the common photoperiod recorded in Wales during the spring-summer season, and also because this photoperiod sustains growth for longer than consistent, 24 h light [41]. During the light phase, carbohydrates are accumulated and metabolized for energy, which is then used in protein synthesis during the dark phase [27].

The growth curves (Figure 4) used optical density measurement at 750 nm as this wavelength reduces absorbance by the pigment chlorophyll, dominant in *C. vulgaris* cells. Across all conditions, the initial growth rate was consistently higher than the overall growth rate. This was due to the high level of nutrients and light availability between day 0 and 2 as a result of low cell density (<22 million cell mL^−1^), which caused high photosynthetic activity, energy production and protein synthesis for cell division [42]. Nutrients were depleted beyond day 2, as shown for PO_4_-P and NH_4_-N (Figure 6 and Figure 7), resulting in reduced assimilation and lower overall growth rates. Growth rates decreased substantially beyond day 7 (Figure 4a–c).

Biomass production was within the expected typical yield of 0.3–0.6 g L^−1^ [43] and biomass productivity was high (>0.16 g L^−1^ d^−1^) under all conditions. T1 and T2 exhibited higher biomass productivity (>0.074 g L^−1^ d^−1^) of *Chlorella* than was obtained in a similar study using autoclaved secondary treated wastewater [23] (Table 4). This was due to the lower level of nutrients in the secondary wastewater used by Cho et al. [23], which contained only 10.0 ± 7.1 mg L^−1^ and 1.7 ± 0.3 mg L^−1^ NH_4_-N and total-P, respectively. In contrast, the biomass production for T1 was far lower than that obtained for *Chlorella* sp. cultivated on autoclaved centrate (1.175 g L^−1^) by Li et al. [26] (Table 4). The centrate contained 10-fold more PO_4_-P, which is used in protein synthesis, than the primary wastewater used in this study and the experiment was conducted over an additional two days. Cho et al. [23] conducted a preliminary experiment with raw untreated effluent. This showed limited growth of *Chlorella* sp. due to the presence of unicellular bacteria and protozoa that feed on microalgae. The presence of these microorganisms can also inhibit microalgal growth by limiting light penetration from the water surface or through accumulating vital nutrients required for the growth of algae [23]. This suggests that pre-treatment of wastewater (using microfiltration, for example), was required for the growth of microalgae and subsequent nutrient removal.

The high levels of initial nutrients imply that the low growth rate in T2 was a result of turbidity due to the presence of colloidal particles and suspended solids [8,23] within the unfiltered wastewater. The presence of these compounds reduced light utilization in photosynthesis, which has been investigated by previous studies [21,23]. The effect of turbidity on algal growth has not yet been concluded, and a study by Wang [45] had similar mixed findings. However, they suggested that the effect of turbidity, either in inhibiting or stimulating growth, could be related to amount of available nutrients in water. In this case, the high levels of nutrients in T2 stimulated growth regardless of the turbidity of unfiltered wastewater. The presence of additional insoluble organic compounds that can be metabolized directly by algal cells [46], may have contributed to the high biomass production in unfiltered wastewater T2. Microalgae have the capacity to improve the environmental conditions of the growth medium by undergoing self-flocculation in order to increase the light permeability in the system and promote growth. Microalgae cultivation shows a capacity of self-adaptation, which is beneficial to wastewater treatment because the water turbidity can change frequently as rainfall and polluting loads vary daily [47]. The effect of 0.2 μm filtration on light availability, through reducing turbidity, may have improved initial growth rates in T1 due to increased light utilization for photosynthesis compared with T2 (Table 4). This suggestion is supported by the results of Cho et al. [23], who found the highest biomass productivity occurred in membrane filtered (0.2 μm) wastewater. However, the present study achieved higher removal efficiencies and higher biomass productivity than Cho et al. [23]. The removal efficiencies obtained by Cho et al. [23] using 0.2 μm membrane filtration were only 92% (T-N) and 86% (T-P) compared to 94% (NH_4_-N) and 97% (PO_4_-P) in the present study. This may be due to the use of primary treated wastewater and a higher intensity of illumination, 177 μmol m^−2^ s^−1^, in the present study compared with secondary treated wastewater and only 60 μmol m^−2^ s^−1^ used by Cho et al. [23] (Table 4).

The control, consisting of F2P media based on the Guillard [48] F/2 medium, had the highest biomass productivity (0.190 ± 0.048 g L^−1^ d^−1^) of all conditions (Table 4). This was an expected result with regard to the growth promoting vitamins [49] presented in F2P, such as thiamine, biotin and cyanocobalamin [50], which were absent from wastewater. However, F2P media consisted of nitrate as the predominant N source and therefore exhibited a lower initial growth rate (0.710 ± 0.034 d^−1^) compared to T1 (0.920 ± 0.050 d^−1^) due to the slower assimilation of nitrate compared with ammonium, which was the predominant N source of domestic wastewater [51,52].

### 4.2. Effect of Wastewater Composition on Nutrient Removal

Freshwater microalgae have been shown to adjust their internal N:P ratio to suit their environment and optimize nutrient uptake and assimilation [42]. This adaptation allows them to exploit the nutrient source provided by wastewater as shown in the high growth rates and biomass production obtained in this study. The optimal inorganic N:P ratio is suggested to be within an ideal range of between 6.8–10 for freshwater algal growth [53,54,55]. Conditions within this study represented N limitation due to the <13 N:P ratio [56]. The optimal inorganic N:P ratio reflects a more widely accepted phytoplankton stoichiometry, the Redfield ratio (106:16:1), which is the average internal composition of C:N:P [57]. The results of this study were found to support the high growth rate and nutrient removal expected from wastewater composition, closely matching the Redfield ratio [33,58]. T1 had an N:P ratio of 7.5:1.0 and exhibited the highest removal efficiency across all conditions (97.69% PO_4_-P). T1 also resulted in higher nutrient removal efficiency of NH_4_-N (94.18%) compared to another study. Wang et al. [44] showed removal of only 82.4% (NH_4_-N) and 83.2% (PO_4_-P) from primary wastewater with a less optimal N:P ratio of 5.9:1.0 (Table 4). Biomass was produced as a result of nutrient uptake and assimilation [59,60,61]. As NH_4_-N regulates lipid content [62], N limitation may have resulted in accumulation of storage lipids within *C. vulgaris* due to the low N:P ratio. Storage lipids are metabolized to release energy under cellular stress [63,64]. Previous work has found microalgae to enhance the secretion of metabolites when cultivated under nitrogen starvation [65]. These additional metabolites contribute to the accumulation of organic matter within the growth medium and have the potential to increase BOD in wastewater effluent. It is, therefore, important to monitor BOD in order to ascertain the optimal level of N limitation with low extracellular organic matter (EOM) [66]. Methods such as cell immobilization have been shown to increase nutrient removal further. Ruiz-Marin et al. [35] reported only 60.1% removal of NH_4_-N from secondary wastewater by free *C. vulgaris* cells compared to removal of 80.0% by cells immobilized in alginate beads (Table 4). 

The lower PO_4_-P removal efficiency of T2 (96.63%) compared to T1 (97.69%) (Table 4) may be due to the lower N:P ratio of T2. N is the limiting factor in P accumulation because N is required in protein synthesis, such as of ribosomal RNA, which incorporates P [33,42]. In the case of N limitation, uptake can still occur through a luxury uptake pathway where polyphosphates accumulate within *C. vulgaris* cells, which hydrolyse these to PO_4_-P [67]. This described effect of N limitation on P removal was observed in a study conducted by Li et al. [26] using a mixture of *Chlorella* sp. cultivated by batch culture. Li et al. [26] found a removal efficiency of only 79% T-P compared to 93% NH_4_-N using autoclaved centrate with an N:P ratio of 1.0:2.1, clearly presenting N limitation.

The composition of wastewater in T1 was most suitable for bioremediation by *C. vulgaris* as this produced the highest initial growth rate and high nutrient removal efficiency of both NH_4_-N and PO_4_-P, comparable to those reported in the literature. Unfiltered wastewater could present a challenge to bioremediation by microalgae when nutrient inputs are low due to the effect of turbidity on light utilization. Previous studies have demonstrated the effect of turbidity on microalgal growth and suggest that phycoremediation techniques should be applied to the wastewater treatment cycle following secondary treatment and settling as water presents more optimal growing conditions in low turbidity [47]. Therefore, with the obtained results and previous research into the application of microalgal bioremediation, it is highly advisable to incorporate a microfiltration step in the wastewater treatment process following secondary treatment, as effluent remains high in inorganic nutrients and has low turbidity. This will achieve high nutrient removal efficiencies.

### 4.3. Mechanisms of Nutrient Removal

The focus of this study was the bioremediation of nitrogen in the form of NH_4_-N and phosphorus in the form of PO_4_-P, as the two main nutrients of concern with regard to eutrophication. The removal of one nutrient depended on the availability of another, as both are required for biomass production [42] hence the variety in removal efficiencies as shown in Table 2.

#### 4.3.1. Nitrogen

Organic N is derived from inorganic sources including nitrate, nitrite and ammonium, which are converted from inorganic to organic form through assimilation by *C. vulgaris*. Inorganic N was translocated across the plasma membrane where it was reduced from nitrate to nitrite and nitrite to NH_4_-N by nitrite reductase and ferrodoxin [61]. NH_4_-N was incorporated into amino acid (glutamine) by glutamine synthase, requiring glutamate and adenosine triphosphate (ATP), for assimilation to peptides, proteins, enzymes and genetic material [33,61].

NH_4_-N was readily assimilated by *C. vulgaris* as it does not require as much energy as other forms of inorganic N such as nitrate [52]. Therefore, nitrate removal does not occur until the majority of NH_4_-N has been consumed [60,68] so wastewaters such as those used in this study, containing high ammonium concentrations, lead to rapid growth of *C. vulgaris* and rapid NH_4_-N uptake, which is a key step in wastewater bioremediation.

#### 4.3.2. Ammonium

*C. vulgaris* growth was accompanied by a decrease in NH_4_-N content of the wastewater due to assimilation of NH_4_-N for biomass production. NH_4_-N is considered the favoured form of inorganic N over nitrate, which may be the reason for the higher initial growth rate in Treatment 1 than the Control (nitrate in F2P media). The differences in growth of *C. vulgaris* between Treatments 1 and 2 could be attributed to the composition of other simple organic N sources such as urea and amino acids, likely to be present in large quantity within domestic wastewater [26]. Additional analysis of the depletion of these organic compounds may have provided better insight into the growth of *C. vulgaris* and possible interactions with removal of the target nutrients, NH_4_-N and PO_4_-P. 

#### 4.3.3. Phosphorous

Organic P is found in nucleic acids, lipids and proteins and plays a vital role in growth and metabolism through its presence in energy transfer compounds such as ATP [61,69,70]. Inorganic P such as PO_4_-P present in the form of H_2_PO_4_^−^ was removed from wastewater by photophosphorylation that occurs within chloroplasts of the chlorophyll present in *C. vulgaris*, producing ATP [71,72]. 

#### 4.3.4. Ortho-phosphate

The removal of PO_4_-P between day 0 and 2 in T1 (Figure 6b) seemed to occur at a higher rate than in T2 (Figure 6c). This could be due to either the lower ratio of N:P or the effect of turbidity on growth in T2. This observation is supported by the higher initial growth rate between days 0 and 2 in T1 (Figure 4b) than T2 (Figure 4c). 

The decline in PO_4_-P below 0.42 mg L^−1^ by day 8 in T1 presents significant difference to T2, which remained above this concentration of PO_4_-P throughout the 12-day growth period. This could explain the lack of death phase in the growth curve for T2 (Figure 5c), where high growth rates occurred as a result of the elevated levels of PO_4_-P (>0.51 mg L^−1^) beyond day 8. The presence of PO_4_-P is likely to be the predominant reason for further growth as NH_4_-N is depleted to <5 mg L^−1^ by day 8 in all conditions. 

Both PO_4_-P and NH_4_-N decreased rapidly in the first 3 days due to fast assimilation by *C. vulgaris.* The same was observed in a similar study using tertiary–treated wastewater [29]. 

### 4.4. Microalgal Uses and Current Technologies

The ability of microalgae to remediate polluting nutrients from wastewater presents an opportunity to reduce the economic and environmental cost of tertiary wastewater treatment whilst improving the quality of final effluent in line with environmental standards (Table 3). Semi-continuous culture can be used for continual removal of nutrients from wastewater by maintaining microalgae in an exponential growth phase [73]. Microalgae can be harvested using a number of methods including centrifugation, sedimentation and flocculation [61]. In certain species of microalgae, the biomass produced by simultaneous bioremediation can itself be applied on land as organic fertilizer either in its raw or semi-decomposed form [74]. This attribute represents the possibility to use microalgae in developing a circular economy [75]. Membrane filtration is also used extensively as a dewatering step in microalgae downstream processing. However, this method could impose a challenge when used in wastewater, as the filter may not separate only microalgal cells from effluent—suspended solids may also accumulate depending on the pore size. This would require maintenance (including chemical cleaning and backwash) and possibly frequent membrane replacements, at a financial cost.

There is some potential for microalgae as feedstock for biofuel metabolites such as lipids and carbohydrates used in the production of biodiesel [76] and other high-value bioactive compounds [77,78]. This could provide solutions to the twin challenges of energy security and environmental pollution [75]. However, as microalgal production requires a large cultivation area and additional energy for harvesting and extraction, the prospects are limited [79]. Research and development (R&D) is needed to develop novel cultivation systems that tackle these issues [79]. Currently, the largest use of the Generally Recognized as Safe (GRAS) *Chlorella* is in human nutritional supplements and food colourings due to antimicrobial [80,81] and antioxidant [82,83] properties. In addition to its use in human nutrition, it plays an important role in the aquaculture industry, unsurprisingly so, as microalgae form a natural food source in freshwater and marine systems [62,84,85].

## 5. Conclusions

The results of this study demonstrate that *C. vulgaris* is well adapted to growth in domestic wastewater and showed high (>95%) removal of NH_4_-N and PO_4_-P from primary filtered wastewater. Although the final effluent levels were classified as moderate-poor with regard to the concentration of nutrients for discharge into water bodies, these results are promising as they present an improvement on the quality of current domestic wastewater effluent from biological filtration. Bioremediation by *C. vulgaris* provides one method for improving current wastewater treatment. The use of membrane technology as a part of the wastewater treatment process enhances the nutrient uptake performance of microalgae and the consequent nutrient removal efficiency, as was demonstrated in this study. The results of this work suggest that growing *C. vulgaris* in nutrient-rich filtered wastewater provides an option for domestic wastewater treatment to improve quality of final effluent.

## Figures and Tables

**Table 1:** Nutrient concentration of autoclaved primary and secondary domestic wastewater from the Southgate facility.

Nutrients	Concentration (mg L^−1^)	
	Primary	Secondary (effluent)
NH_4_-N	104.51 ± 0.455	<5 ^1^
PO_4_-P	23.65 ± 0.087	15.41 ± 0.066

^1^ Reference to a level below 5 mg L^−1^, as this is the detection limit for NH_4_-N.

**Table 2:** Characteristics of growth media before (initial) and after (final) the 12-day growth period of *C. vulgaris*.

		Control	Treatment 1	Treatment 2
Initial	N:P	2.4:1.0	7.5:1.0	4.4:1.0
	NH_4_-N (mg L^−1^)	5 ^1^	85.84	104.51
	PO_4_-P (mg L^−1^)	2.47	13.54	23.65
Final	NH_4_-N (mg L^−1^)	5 ^1^	5 ^1^	5 ^1^
	PO_4_-P (mg L^−1^)	0.256	0.313	0.796
% removed	NH_4_-N	NA	94.18%	95.22%
	PO_4_-P	89.64%	97.69%	96.63%

^1^ Reference to a level at or below 5 mg L^−1^, as this is the detection limit for NH_4_-N.

**Table 3:** Classification of the quality of wastewater following bioremediation by *C. vulgaris* compared to current wastewater treatment.

Measure of Effluent Quality	Nutrient/Discharge Area	Treatment 1	Treatment 2	Current ww Treatment (Table 1)
Final level	NH_4_-N mg L^−1^	<5	<5	<5
	PO_4_-P mg L^−1^	0.313	0.796	15.41
Classification	Rivers	Moderate-Poor	Moderate-Poor	Poor
	Coastal	Poor	Poor	Poor
	Lakes	<Good	<Good	<Good

**Table 4:** Summary of results and comparison with previous studies.

Strain	Culture Medium/Wastewater	Pre-Treatment	Light Intensity (μ mol m^−2^ s^−1^)	µ_1_ (d^−1^)	% NH_4_-N	% PO_4_-P	Biomass Production (mg L^−1^)	References
*C. vulgaris*	Primary (T1)	0.2 μm filtration	177	0.92	94.18	97.69	0.513	This work
*Chlorella sp.*	Primary	filtration	200	0.43	82.40	83.20	-	Wang et al. [44]
*Chlorella sp. 227*	Secondary	0.2 μm filtration	60	-	92.00	86.00	-	Cho et al. [23]
*Chlorella sp. 10b*	Centrate	-	50	0.48	93.00	79.00	1.175	Li et al. [26]
*C. vulgaris*	Secondary	-	135	0.19	60.10	-	-	Ruiz-Marin et al. [35]
*C. vulgaris*	Primary (T2)	-	177	0.39	95.22	96.63	0.527	This work
*C. vulgaris*	F2P media	-	177	0.71	-	89.64	0.457	This work
